# Cardiac lymphoma with early response to chemotherapy: A case report and review of the literature

**DOI:** 10.1007/s12350-021-02570-5

**Published:** 2021-03-11

**Authors:** Andrea Bonelli, Sara Paris, Stefano Bisegna, Giuseppe Milesi, Emanuele Gavazzi, Raffaele Giubbini, Chiara Cattaneo, Fabio Facchetti, Pompilio Faggiano

**Affiliations:** 1 Cardiology Unit, Spedali Civili and University of Brescia, Piazza Spedali Civili, Brescia, Italy; 2 Department of Radiological Sciences, Spedali Civili and University of Brescia, Piazza Spedali Civili, Brescia, Italy; 3 Nuclear Medicine Unit, Spedali Civili and University of Brescia, Piazza Spedali Civili, Brescia, Italy; 4 Hematology Unit, Spedali Civili and University of Brescia, Piazza Spedali Civili, Brescia, Italy; 5 Pathological Anatomy Service, Spedali Civili and University of Brescia, Piazza Spedali Civili, Brescia, Italy

**Keywords:** Basic science, modalities, diseases/processes, technical

## Abstract

Cardiac tumors are rare and benign masses account for the most part of the diagnosis. When malignant cancer is detected, primary or secondary cardiac lymphoma are quite frequent. Cardiac lymphoma may present as an intra or peri-cardiac mass or, rarely, it may diffusely infiltrate the myocardium. Although often asymptomatic, patients can have non-specific symptoms. Acute presentations with cardiogenic shock, unstable angina, or acute myocardial infarction are also described. Modern imaging techniques can help the clinicians not only in the diagnostic phase but also during administration of chemotherapy. A multidisciplinary counseling and serial multi-parametric assessment (echocardiography, cardiac troponin) seem to be the most effective approach to prevent possible fatal complications (i.e., cardiac rupture). Currently, only chemo- and radiotherapy are available options for treatment, but the prognosis remains poor. This is a case of secondary cardiac lymphoma presenting as a mediastinal mass with large infiltration of the heart and the great vessels with a good improvement after only one cycle of chemotherapy. It demonstrates the importance of an early diagnosis to modify the natural history of the disease.

## Introduction

Malignant neoplasm, such as sarcomas and primary lymphomas, is very rare. In fact, almost three out of four cardiac masses are benign tumors.^1^ However, metastatic invasion of the heart is quite frequent.^2^

Cardiac involvement in case of lymphoma is commonly clinically silent and often discovered *post-mortem*.^2^ Nonetheless, some patients may complain symptomatic manifestations.^2^,^3^

Although the modern combination schemes of chemotherapy, the general prognosis of primary or secondary cardiac lymphomas is usually poor, partly because of diagnostic delay.^3^,^4^ So, it is crucial to suspect and detect early cardiac involvement, especially with the support of imaging techniques. Echocardiography is the method of choice to detect cardiac involvement and complications.^5^

In this case report, we present an unusual case of massive cardiac infiltration due to mediastinal lymphoma, where the multidisciplinary approach in the diagnostic phase and during the first cycle of chemotherapy has been of primary relevance. The close imaging follow-up and the efficacy of hematologic therapies have been crucial to avoid potential life-threatening complications.

## Case Report

On May 2020, a 54-year-old man came to our attention in order to undergo surgical ablation of persistent atrial fibrillation. He was symptomatic for palpitation, dyspnea, and mild chest oppression.

During the previous year, the patient had multiple episodes of atrial fibrillation and he was also hospitalized for pericardial effusion causing cardiac tamponade and for intraparenchymal cerebral hemorrhage which necessitated surgical evacuation. In that occasion, the pericardiocentesis revealed hematic fluid with bacterial growth, so he was treated with antibiotic therapy.

**Figure 1:**
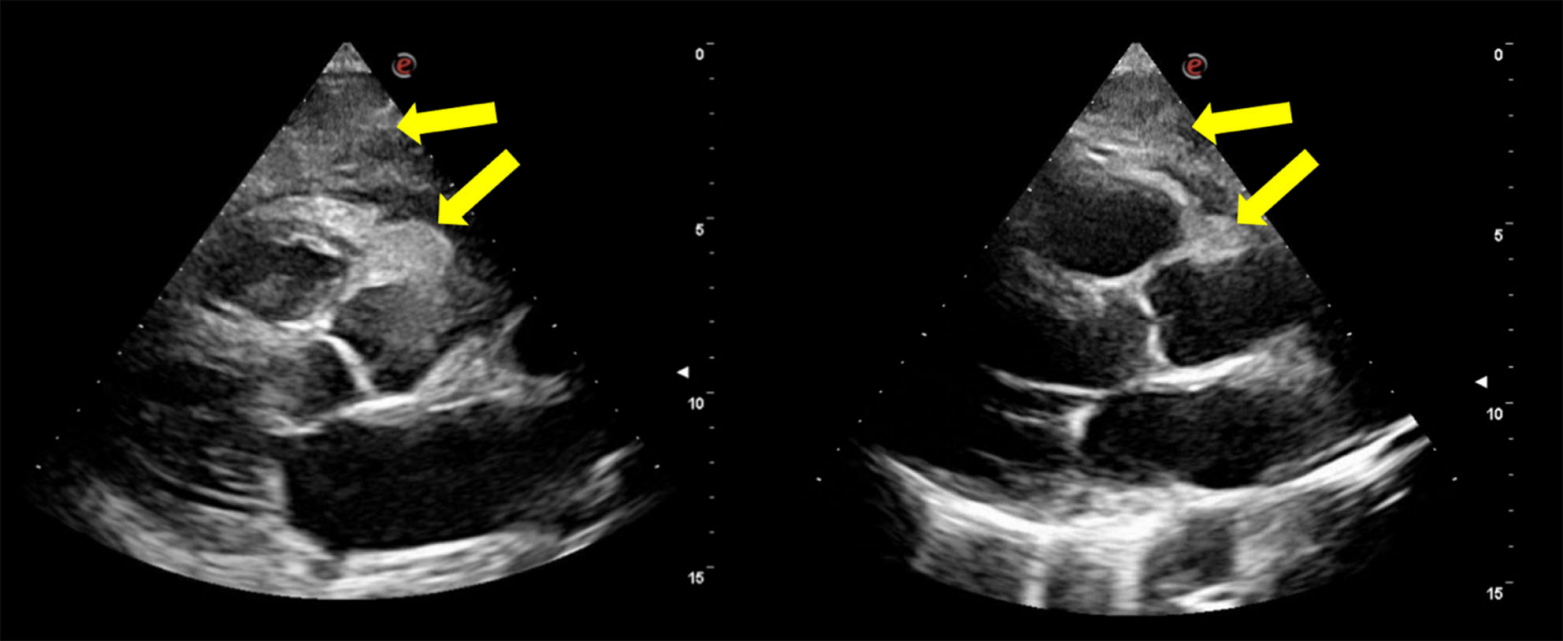
Trans-thoracic echocardiography - parasternal long-axis view. On the left, it is evident the marked and diffused thickening of the myocardium, especially of the interventricular septum, the posterior, the interatrial septum and the right ventricular free wall, which appear hypokinetic. Also, the large iso-echogenic mass takes up space around the ascending aorta and in the anterior mediastinum (yellow narrow). On the right, the view after the first chemotherapy regimen

Before undergoing surgical ablation, the trans-thoracic echocardiogram pointed out a marked and diffused thickening of the myocardium and a large iso-echogenic peri-cardiac mass as well as sign of pulmonary and systemic congestion, mild pericardial effusion, and severe left pleural effusion (Figure 1).

**Figure 2:**
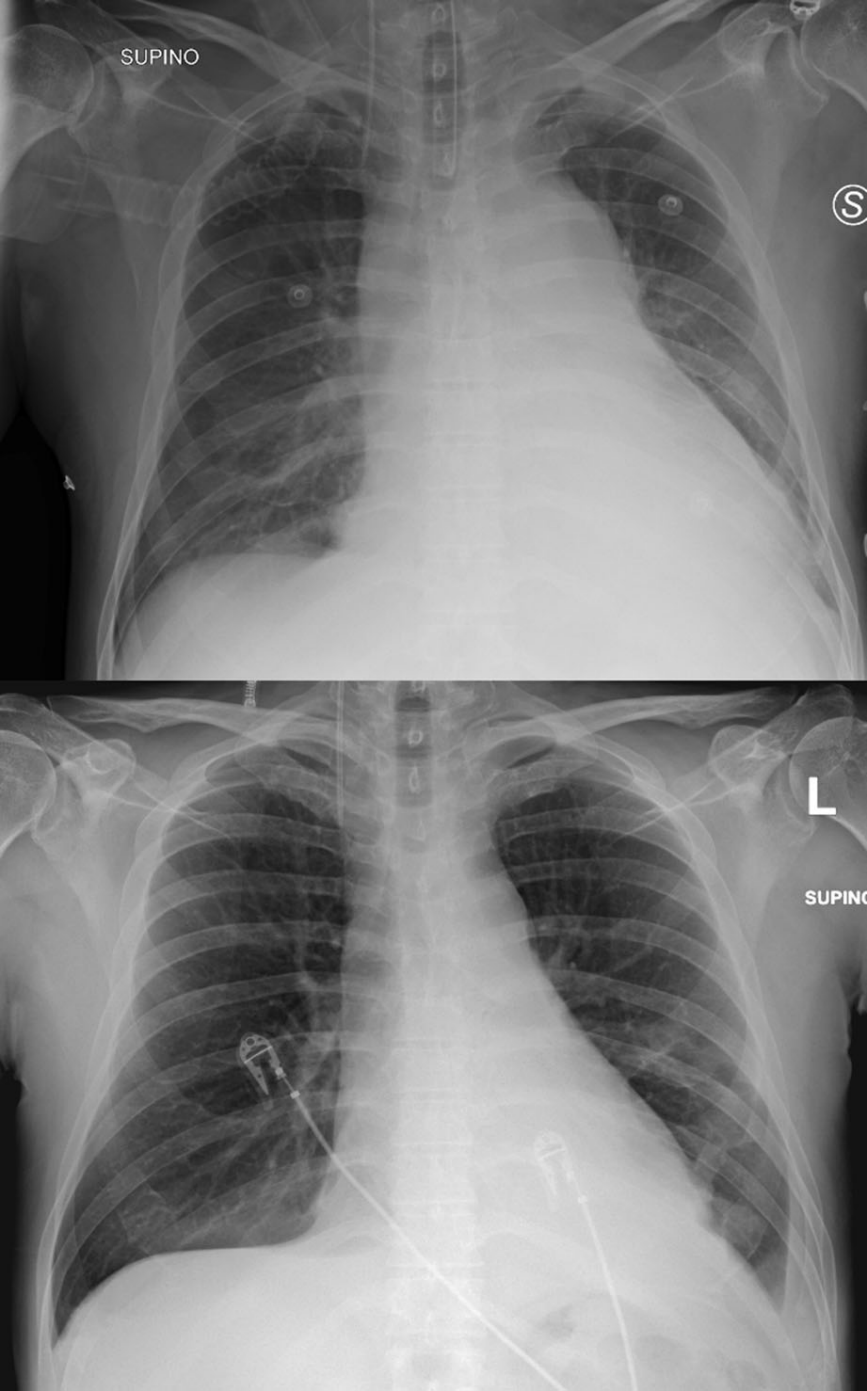
Chest X-ray - The upper picture shows the parenchymal thickening of the left inferior pulmonary lobe with associated pleural effusion. Note the enlarged mediastinum and the increased dimension of the heart. The inferior picture shows the chest X-ray after the first cycle of chemotherapy, with net reduction of the pleural effusion and the mediastinal mass

The chest X-ray and the computed tomography confirmed the presence of an abundant solid tissue in the anterior mediastinum, surrounding the great vessels. Multiple enlarged lymph nodes above and below the diaphragm were also described (Figure 2).

**Figure 3:**
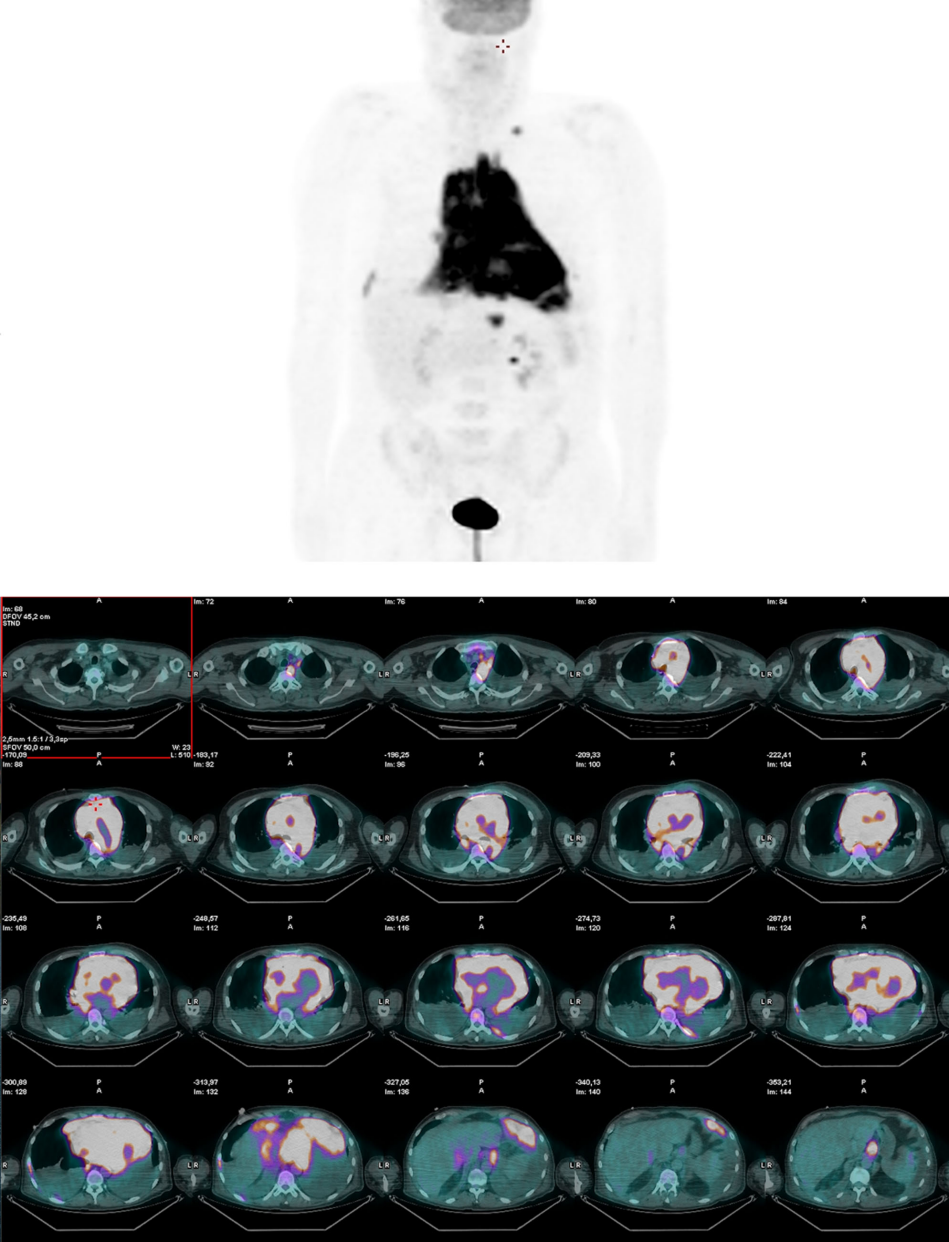
[18F]-Fluorodeoxyglucose positron emission tomography. Global and diffused accumulation of glucose in the mediastinum and in the heart, with the involvement of both ventricles, interventricular septum, and atria. Subdiaphragmatic lymph nodes were described too

To complete the evaluation, positron emission tomography (PET) was performed and confirmed subdiaphragmatic lymph nodes and global accumulation of 18-FDG in the heart (Figure 3).

**Figure 4:**
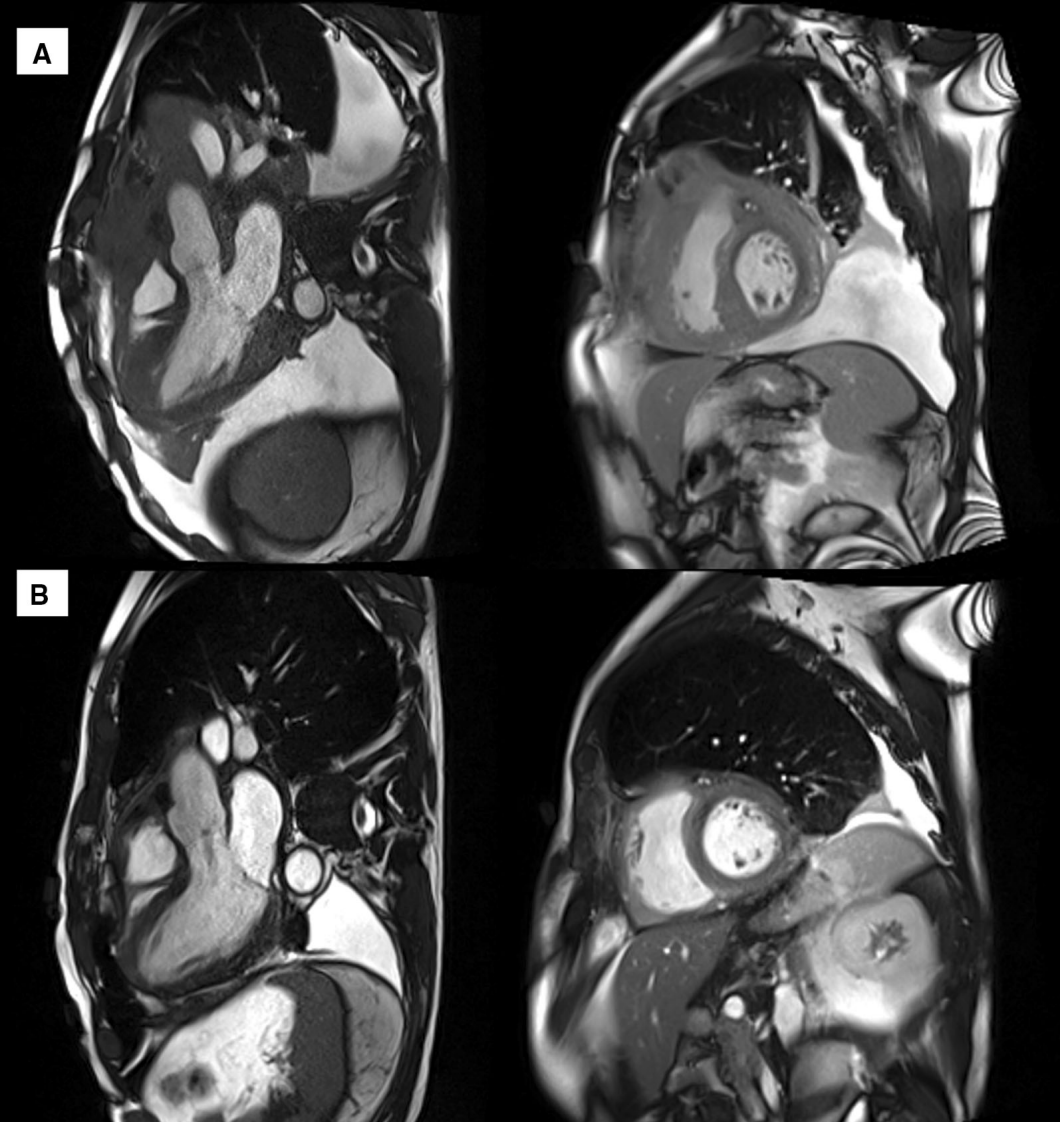
Cardiac magnetic resonance. **A** 3-chamber view and mid-ventricular short-axis view before chemotherapeutic treatment. Note the massive invasion of the heart by the mediastinal mass, whose cleavage borders are not evaluable; the myocardium appears thickened. Diffuse edema in T2-weighted sequences was detected, with a similar diffuse LGE pattern. **B** After the first regimen of chemotherapy, no more myocardial edema and late gadolinium enhancement were noticed, with near-normalization of left ventricle thickness

Also, the cardiac magnetic resonance (CMR) demonstrated massive invasion of the heart by the mediastinal mass, whose cleavage borders were not evaluable. Marked thickening of the myocardium with sign of diffuse edema on T2-mapping and the presence of late gadolinium enhancement were found (Figure 4)

**Figure 5:**
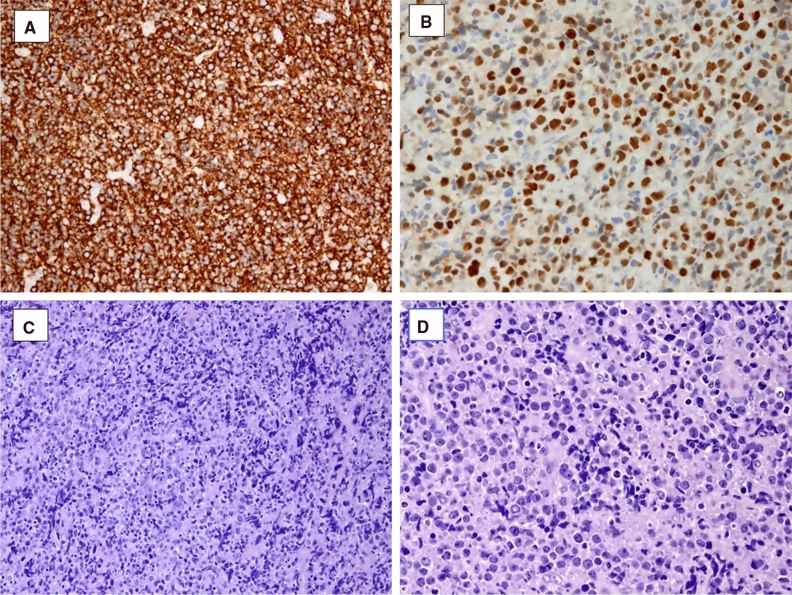
Tumor histology. High-grade diffuse large B-cell lymphoma with expression of CD20 (**A**) and BCL-6 (**B**). The hematoxylin–eosin stain (**C** and **D**) shows medium to large cells with irregular nucleus

The bone marrow biopsy did not find any localization of the malignancy, thus the thoracoscopic biopsy of the mediastinum was performed. The histological examination confirmed the diagnosis of diffuse large B-cell lymphoma (Figure 5). Thus, according to hematological indications, a chemotherapy regimen was started. Given the massive cardiac infiltration, according to the hematologist consultation, the first dose of the R-CHOP cycle (rituximab, cyclophosphamide, doxorubicin, vincristine, and prednisone) was administered in three stages. During the administration, echocardiogram was performed every 48 hours for the first 10 days, in order to detect early occurrence of complications, such as pericardial effusion or cardiac rupture.

The patient improved quickly. On day 7 after the first cycle of chemotherapy, dyspnea and chest oppression disappeared, as well as the signs of systemic congestion, together with concomitant significant reduction of the mediastinal mass and complete resolution of the pleural effusion.

**Figure 6:**
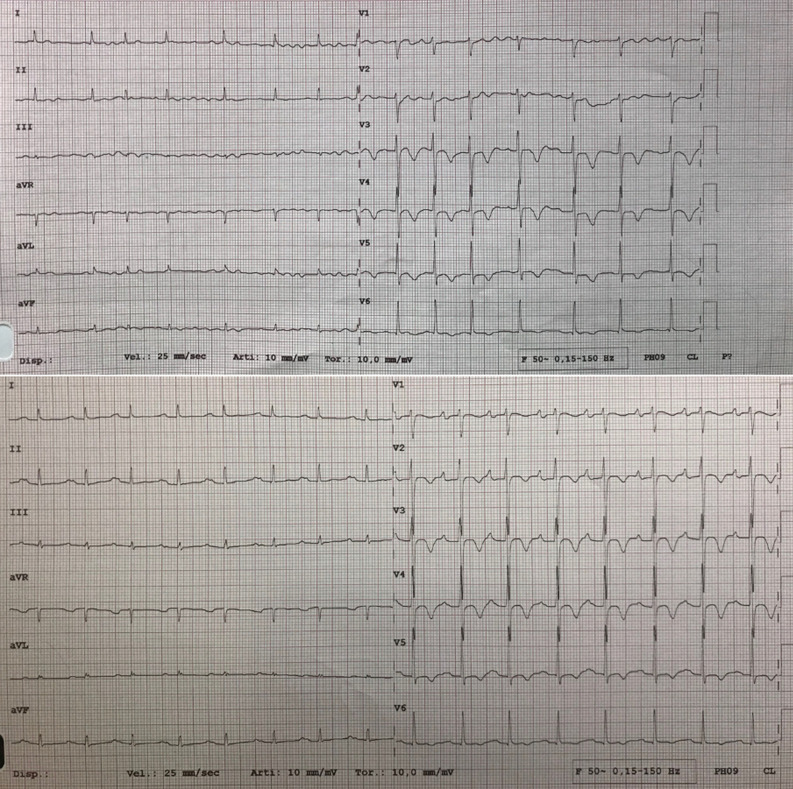
Electrocardiogram before and after the first cycle of chemotherapy showing atrial fibrillation and subsequent spontaneous restoration of sinus rhythm. Note the negative T waves on the precordial leads

Lastly, there was a spontaneous restoration of sinus rhythm and a heart rate reduction (Figure 6).

Despite the rapid improvement of the cardiac infiltration, after 5 months of chemotherapy, the patient had a progression of the disease with cerebral localization, so he was addressed to palliative cares.

## Discussion

This case reflects many features of the secondary cardiac lymphoma.

Primary cardiac lymphoma (PCL) is a non-Hodgkin lymphoma which involves primarily the heart and/or the pericardium, with the bulk of the tumor intrapericardial.^6^ Unlike PCL, secondary cardiac localization accounts for 25% of all disseminated lymphoma.^3^

The most prevalent secondary lymphomas are B-cell origin non-Hodgkin lymphomas, and diffuse large B-cell is the most frequent (80%). It usually affects immunocompetent male patients, with a median age of 60 years old.^7^ In case of immunocompromised individuals, cardiac lymphoma is usually virus-related (Epstein–Barr virus, human herpes virus-8) .^3^

On the other hand, cardiac involvement from Hodgkin lymphoma has been rarely detected.^2^

Typically, both primary and secondary cardiac lymphomas can be infiltrative, intramural, or may appear as epicardial lesions, singular or multiple, with a particular tropism for the pericardium and the right side of the heart.^3^ Usually cardiac valves are spared.^5^

Sometimes, cardiac involvement in disseminated lymphoma is widespread, affecting not only the pericardium but also the myocardium, diffusely.^8^ Some case reports showed uncommon presentations like massive cardiac infiltration mimicking restrictive cardiomyopathy.^6^,^8^–^10^

The localization of the tumor reflects the way of its spread. It can be lymphatic, hematogenous or by direct extension.^8^ A triple-way diffusion is possible, especially in case of widespread lymphoma.

Although it is usually detected during autopsy, non-Hodgkin lymphoma may be clinically evident and lead to potentially lethal complication.

Signs and symptoms depend on several factors, such as tumor location, size, growth rate, degree of invasion, and friability.^4^ Many of the symptoms are non-specific, particularly if the metastasis is not extensive.^2^ The most common symptom is dyspnea, followed by chest pain and B-symptoms (i.e., weight loss, fatigue, night sweats). Congestive heart failure with peripheral edema and pulmonary congestion characterizes 50% of clinical presentation. Cardiogenic shock, sudden cardiac death, acute myocardial infarction, and myocardial perforation have also been reported (Table 1).^11^

**Table 1:** Summary of case reports of primary and secondary cardiac lymphoma

Reference, year	Histology	Localization in heart	Symptoms	Diagnostic tool	Therapy	Outcome
Wiernick et al. (1976)^25^	Lymphocytic lymphoma	Infiltration of the lateral wall of the left ventricle	Acute myocardial infarction Heart failure	Autopsy	Chemotherapy Radiotherapy	9 years survival
Collazzo et al. (1987)^26^	Lymphoblastic lymphoma	Cardiac apex	None	TTE	Local radiotherapy	12 months survival
	Burkitt’s lymphoma	Epicardium, posterior ventricular wall and interventricular septum Pericardial	Dyspnea	TTE	Local radiotherapy	20 days survival
Cracowski et al. (1997)^27^	High-grade malignant lymphoma	Left ventricular hypertrophy Increased echogenicity of the myocardial walls Reduced ejection fraction	Dyspnea	TTE Endomyocardial biopsy	Chemotherapy	NA
Beckwith et al. (2000)^21^	Diffuse large B-cell type lymphoma	PCL Right atrial mass	Dyspnea Cough Fatigue Weight loss	TEE	Chemotherapy (half-dose regimen) Radiotherapy Pericardial patch	2 months survival
Cho et al. (2002)^28^	Diffuse large B-cell type lymphoma	Interventricular septum and left ventricular posterior wall	Dyspnea Palpitation	TTE	Chemotherapy	NA
Bergler-Klein et al. (2003)^29^	Burkitt’s lymphoma	Asymmetric hypertrophy of the mid and distal septum Speckled appearance of the myocardium and LV apical region	None	TTE CMR PET	Chemotherapy	9 months survival
Dawson et al. (2006)^30^	Diffuse large B-cell type lymphoma	PCL Extensive intramyocardial mass involving the right side of heart and around the pulmonary artery trunk	Dyspnea Syncope	TEE CMR CT PET	Chemotherapy (half-dose regimen)	Alive after 11 months
Shah et al. (2014)^23^	Diffuse large B-cell type lymphoma	PCL Large right atrial mass with in severe tricuspid stenosis and surrounding the aortic root	Weight loss Fatigue Night sweats	TTE TEE PET	Low-dose chemotherapy regimen followed by full dose regimen	In remission after 12 months
Jonavicius et al. (2015)^31^	Diffuse large B-cell type lymphoma	PCL Left and right atrium Left ventricle through interventricular septum	Heart failure Dyspnea Cyanosis B-symptoms	TTE Open chest biopsy	Chemotherapy	NA
	Diffuse large B-cell type lymphoma	Large mass in the right heart, obstructing right ventricle Pericardial effusion	Heart failure Chest pain	TTE CMR Percutaneous trans-venous biopsy Open chest biopsy	Palliative surgery (Fontan procedure)	Died during surgery
Cereda et al. (2017) (three patients report)^24^	Diffuse large B-cell type lymphoma	PCL Right side of heart	NA	TTE PET CT	Chemotherapy	In remission after 25 months
Sarr et al. (2017)^32^	T-cell lymphoma	PCL Right atrial mass Obstruction of tricuspid valve	Right heart failure Cardiogenic shock	TTE CT	NA	Died at admission
Cheng et al. (2018)^33^	Diffuse large B-cell type lymphoma	PCL Right atrial mass hypertrophic left ventricular posterior lateral wall Impaired left ventricular contractility Severe mitral regurgitation Hemopericardium	Acute heart failure Cardiogenic shock Sudden cardiac death resuscitated	TTE CMR Endomyocardial biopsy Open chest biopsy	Chemotherapy (reduced dose)	Died after 2 weeks
Al-Mehisen et al. (2019)^8^	Diffuse large B-cell type lymphoma	Large mass invading both atria and interatrial septum Occlusion of pulmonary veins Compression of right pulmonary artery	Dyspnea Orthopnea Dry cough	TTE Strain CT CMR	Chemotherapy (specific reduced-dose regimen)	In remission after 3 years
Bonou et al. (2019)^34^	Diffuse large B-cell type lymphoma	PCL Heterogeneous mass in the right atrium, infiltrating the interatrial septum Occlusion of the superior vena cava	Dyspnea Jugular vein dilation	TTE TEE CMR CT PET Endomyocardial biopsy	Chemotherapy (initial low-dose regimen)	Alive after 15 months
Oder et al. (2020)^12^	Diffuse large B-cell type lymphoma	Mild left ventricular hypertrophy Right and circumflex coronary artery stenosis	Unstable angina Troponin elevation Third-degree atrioventricular block	TTE IVUS	Percutaneous angioplasty (circumflex artery) Chemotherapy	NA
Bonelli et al. (present case)	Diffuse large B-cell type lymphoma	Massive infiltration of the heart (left and right ventricle, interatrial septum) Thickening of the myocardium Surrounding the ascending aorta Pericardial effusion	Dyspnea Palpitation Mild chest oppression	TTE CMR CT PET Mediastinal biopsy	Chemotherapy (initial half-dose regimen)	After initial improvement, progression of the lymphoma to the brain and started palliative cares

*CMR*, cardiac magnetic resonance; *CT* computed tomography; *IVUS* intravascular ultrasound; *NA* not available; *PET*, positron emission tomography; *PCL* primary cardiac lymphoma; *TTE* trans-thoracic echocardiography, *TEE*, trans-esophageal echocardiography

Due to its location in the right or left heart, a tumor embolization in the pulmonary or the systemic system can, respectively, occur.^3^

Direct infiltration or compression of coronary arteries is rare.^12^ If present, patients can experiment angina, also with troponin elevation (Table 1). Our patient had CT finding of epicardial involvement of coronary arteries too, but clinically silent.

The lymphomatous cells can infiltrate the electrical conduction system of the heart and determine electrocardiographic (ECG) abnormalities. Frequently, these include atrial arrhythmias and atrioventricular blocks. Other ECG alterations descripted are right bundle branch block, inverted T waves, low voltage, as well as life‐threatening ventricular tachyarrhythmia.^13^ The risk of sudden cardiac death is present. In particular, T-cell lymphomas seem to be more related to sudden cardiac death when compared to B-cell ones, because of a highest predisposition to infiltration.^5^

ECG as well as chest X-ray is not sensitive tool to recognize or suspect cardiac infiltration.^14^

In case of suspected cardiac involvement, trans-thoracic echocardiography is the first-line imaging modality.^3^ The typical features include right or left ventricular hypertrophy, and hypo- or akinetic areas where the infiltrative tumor grows. The infiltration can extend and modify epicardial and endocardial borders, as well as surround the peri-cardiac vessels.^5^ When the pericardium is involved, lymphomas may cause pericardial effusion, particularly hemorrhagic effusion, and cardiac tamponade.^3^

Our case is a rare presentation of a massive infiltration of both ventricles, with extension to the peri-aortic space. Only other few case reports displayed similar findings, with a recall to infiltrative-hypertrophic cardiomyopathy.^6^,^9^,^10^

Trans-esophageal echocardiography is an imaging technique potentially useful in these patients, but risky, due to the contiguity between the esophagus and the pericardium sac and the heart. Accordingly, even in our case, this approach was avoided.

Thanks to its tissue resolution, computed tomography (CT) can demonstrate the morphology, the location, and the extension of cardiac or mediastinal mass. The usual findings are single or multiple hypo- or iso-attenuating, variably enhancing mass.^8^ However, myocardial infiltration may be detected with CT (i.e., hypertrophic left ventricular septum). One of the disadvantages is the absence of real-time images.^3^ CT is the gold standard to stage the disease.

The preferred imaging modality is cardiac magnetic resonance (CMR) because of better temporal and spatial resolution. The variable signal intensity is due to the cellularity of the neoplasm: tumor cells have a higher free-water content, so they commonly appear hypointense on T1-weighted MR sequences and hyperintense in T2. On contrast, late gadolinium enhancement (LGE) does not show specific pattern for cardiac lymphoma presenting as a mass (commonly minimal or no enhancement).

Finally, [18F]-Fluorodeoxyglucose positron emission tomography, biopsy of the bone marrow or, directly, of the mass, completes the evaluation and confirms tumor extension. Moreover, PET has recently been reported to detect clinically silent cardiac involvement.^4^

Despite the improvement of imaging tools, cardiac lymphoma has still a diagnostic delay because of a low index of suspicion and a rapid evolution.^6^Thus, the prognosis remains poor for both primary and secondary cardiac lymphoma. Four risk factors for a worse survival are: being immunocompromised, the presence of extracardiac disease, left ventricular involvement, and absence of arrhythmia.^3^,^8^ Usually, heart failure and sepsis are the common cause of death. Gordon et al. showed that the initial presentation had some impact on prognosis, with a median survival of 3 months.^2^ In general, the mean survival is about 12 months after diagnosis.^3^ However, other case series also displayed better survival thanks to modern chemotherapy schemes.^8^

No guidelines are available regarding treatment.^4^ The only effective therapy is chemotherapy and, in many cases, it has only palliative purposes.^5^ The therapeutic schemes include different combination of drugs, where the most used is CHOP (cyclophosphamide, doxorubicin, vincristine, and prednisone). Rituximab and monoclonal therapies are often administered.^4^ Usually, cardiac infiltration is sensitive to chemo- and radiotherapy.^15^ However, radiation is confined to cardiac masses that progress despite chemotherapy, because of cardiovascular side effects.^4^ In some case series, it is reported that the overall response rate of primary cardiac lymphoma to chemotherapy is 79% and complete remission is 59%. Similar results are shown for secondary lymphoma.^16^

Eventually, surgical excision is only addressed to exceptional cases (i.e., patient with heart valve obstruction and good estimated prognosis).^5^

Two points appear to be fundamental: early administration of chemotherapy and prevention of complication. Although rare, some fatal events may occur during the initiation of chemotherapy.^4^,^8^ Ventricular fibrillation, refractory heart failure, myocardial injury, and massive pulmonary embolism may complicate the management.^17^ Moreover, a theoretical risk of cardiac rupture is also reported, particularly in case of rapid tumor destruction. In fact, diffuse large B-cell lymphoma is also known to potentially cause perforation of hollow organs (i.e., trachea and gastro-intestinal tract).^18^ Evidences from several case reports or retrospective case series show a dose reduction regimen or a chemotherapy delay to be effective in reducing complications (Table 1).^8^ However, because of the lack of strong recommendations, an individualized approach, based on clinical suspicion or concern, should be used^19^. A prophylactic placement of pericardial patch over the atrial free wall to protect against rupture has been described^20^–^24^.

In order to detect early complications, a multidisciplinary approach, involving different physicians (i.e., cardiologist, hematologist, cardiac surgeon), is suggested. The role of cardiovascular imaging is crucial as well as serial troponin measurement.

## New Knowledge Gained

This review confirms that multimodal imaging assessment is crucial to diagnose cardiac lymphoma and to early detect the occurrence of complications. Since its suspect, cardiac lymphoma necessitates multidisciplinary interventions.

## Conclusion

Primary cardiac lymphoma is a rare condition, while metastatic localization of lymphoma is more frequent.^3^ Although usually clinically silent, some non-specific symptoms may occur. In particular, our patient had electrical disturbances and sign and symptoms of congestion.

Multimodality imaging is helpful and confirmation with biopsy is mandatory. Once detected, the cardiac lymphoma necessitated early administration of chemotherapy.

The risk of complications during chemotherapy cycles is low, but still present. Serial echocardiographic testing, electrocardiographic monitoring, and evaluation of biomarkers (i.e., cardiac troponin) may help the clinician to find out complications, especially in the very first phases. It seems that a low-dose regimen, especially in the initial phases, may be safe and effective in the prevention of complications.

Early response to the drugs seems to be not infrequent, with relief of the symptoms. However, despite modern effective chemotherapy regimens, overall prognosis remains poor.

## Abbreviations

CMR: Cardiac magnetic resonance
CT: Computed tomography
ECG: Electrocardiogram
IVUS: Intravascular ultrasound
LGE: Late gadolinium enhancement
NA: Not available
PCL: Primary cardiac lymphoma
PET: Positron Emission Tomography
TEE: Trans-esophageal echocardiography
TTE: Trans-thoracic echocardiography
